# In this month’s Bulletin

**DOI:** 10.2471/BLT.17.000417

**Published:** 2017-04-01

In the editorial section, Hajime Inoue & Ren Minghui (242) advocate for immediate national action on antimicrobial resistance. Katherine Littler et al. (243) review progress on data sharing in public health emergencies.

Claire Keeton (246–247) reports on how social innovation can improve access to health care in low-income countries in Africa. Gary Schwitzer (248–249) explains to Fiona Fleck how he seeks to improve media coverage of health topics. 

## Nepal 

### How equitable are vaccine distributions?

Ashish KC et al. (261–269) analyse immunization coverage and patterns from 2001–2014. 

## Ethiopia

### Toilets and trachoma

William E Oswald et al. (250–260) quantify associations between community sanitation use and children with active trachoma.

## Peru

### Incentives to continue tuberculosis treatment

Tom Wingfield et al. (270–280) randomize households to test the effects of socioeconomic support.

## Bangladesh, Ethiopia, Guinea, Haiti, India, Malawi, Nepal, South Sudan, Thailand

### Dealing with cold-chain logistics, regulatory issues and vaccine uptake

Amber Hsiao et al. (303–312) describe 12 cholera campaigns in 9 countries.

## Barbados, Democratic Republic of Congo, Egypt, Gambia, Ghana, Lao People’s Democratic Republic, Malawi, Mongolia, Nepal, Nigeria, Pakistan, Papua New Guinea, Senegal, Sierra Leone, Uganda. 

### Turning on hospital oxygen 

Hamish Graham et al. (288–302) analyse what’s needed to improve oxygen delivery to paediatric patients. 

## Global

### Surviving a mother’s death

Lana Clara Chikhungu et al. (281–287) review the evidence for links between child and maternal mortality.

